# Validation of structural and functional lesions of diabetic retinopathy in mice

**Published:** 2010-10-19

**Authors:** T.S. Kern, J. Tang, B.A. Berkowitz

**Affiliations:** 1 Case Western Reserve University, Cleveland, OH; 2 Veterans Affairs Medical Center, Cleveland, OH; 3 Wayne State University, Detroit, MI

## Abstract

Diabetic retinopathy is a serious long-term complication of diabetes mellitus. There is considerable interest in using mouse models, which can be genetically modified, to understand how retinopathy develops and can be inhibited. Not all retinal lesions that develop in diabetic patients have been reproduced in diabetic mice; conversely, not all abnormalities found in diabetic mice have been studied or identified in diabetic patients. Thus, it is important to recognize which structural and functional abnormalities that develop in diabetic mice have been validated against the lesions that characteristically develop in diabetic patients. Those lesions that have been observed to develop in the mouse models to date are predominantly characteristic of the early stages of retinopathy. Identification of new therapeutic ways to inhibit these early lesions is expected to help inhibit progression to more advanced and clinically important stages of retinopathy.

## Introduction

Studies in diabetic animals have provided valuable insight into the pathogenesis of diabetic retinopathy (DR). For example, studies of diabetic dogs demonstrated that improved glycemic control could inhibit the development of the retinopathy more than 16 years before a comparable demonstration in diabetic patients [1]. The possibility of genetic manipulation, and the availability of reagents and antibodies for molecular studies, has led to great interest in smaller species such as the mouse for studies of DR. In addition, the development of noninvasive, translational imaging methods that can be applied to both humans and animals has opened new opportunities for investigating the pathophysiology of DR. Several laboratory species have been compared with regard to which retinal lesions develop in diabetes, with the general conclusion that more types of lesions develop in larger, longer-lived models (primates, dogs) than in smaller, shorter-lived models (rats, mice) [2].

At present, a range of anatomic and functional retinal lesions linked with DR have been identified by a variety of methods. The identification of the lesions that are most critical to this retinopathy, as well as the methods that best demonstrate these abnormalities, has been controversial. In the present text, “robust” lesions are defined as those that occur in both human and mouse models, and have been identified by different laboratories, independent of the methods used. This category does not rule out the possibility that other retinal lesions represent real and important damage inflicted on the retina by diabetes, but indicates only that important inconsistencies and/or questions remain in the literature. To aid future efforts at translational studies, in this review, we will emphasize existing anatomic and physiologic lesions that fit this classification.

## 1. Diabetic retinopathy in humans

DR is a common complication of diabetes, and one of the leading causes of visual loss in working age populations in developed countries. It has been found to affect the majority of patients who have had diabetes for 1–2 decades, although not all patients develop a comparable severity of retinal disease. The major risk factors of DR are the degree and duration of hyperglycemia. The retinal lesions that develop in type 1 diabetes are not different from those that develop in type 2 diabetes, although the severity and/or incidence of the lesions may differ. For example, the severity of retinal edema and neovascularization differ between different types of diabetes [3].

### 1A. Characteristic structural changes

#### 1A1. Vascular pathology

Clinically detectable characteristics of DR have focused on damage to the retinal vasculature. Based on vascular changes, DR is subdivided into an early nonproliferative stage including progressive capillary occlusion and degeneration (NPDR), and a more advanced, proliferative or neovascular stage (PDR) [4]. In addition, macular edema (i.e., retinal edema involving or threatening the macula) can occur in both stages, can be diffuse or focal in distribution, and can have a cystoid appearance or not.

**Figure 1:**
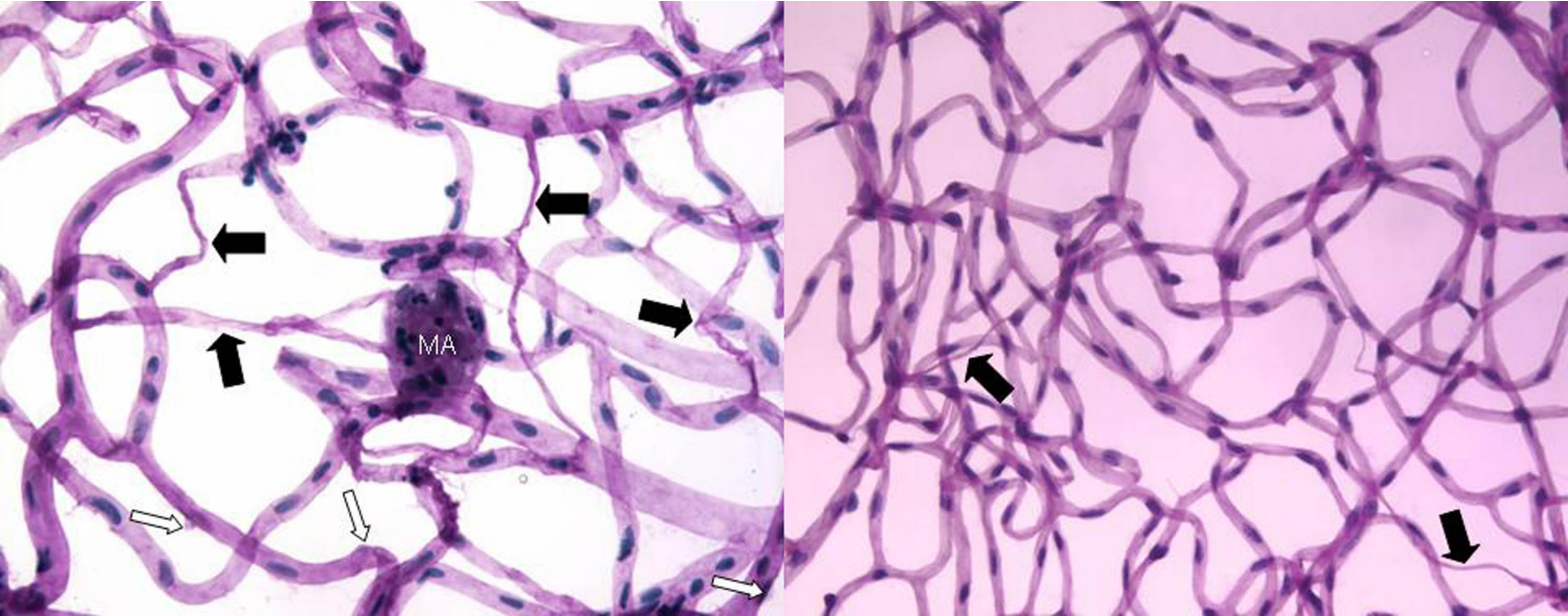
Demonstration of clinical and experimental diabetes-induced degenerate capillaries. Black arrows are diabetes-induced degenerate capillaries and pericyte ghosts are shown in white arrow, and capillary microaneurysm (MA) in isolated retinal vessels. **A**: A diabetic patient having non proliferavtive diabetic retinopathy (NPDR). **B**: A C57Bl/6 mouse after nine months of diabetes. Pericyte ghosts are more difficult to detect in mice retinas than in most other species.

Other vascular lesions of NPDR include the appearance of microaneurysms, vascular nonperfusion, and degeneration (Figure 1). Capillary degeneration and nonperfusion are tightly associated, but it remains unclear if they occur independently of each other, or if one always causes the other. Nonetheless, degenerate capillaries are very important lesions of the retinopathy; they lead to progressive reductions in retinal perfusion, at least locally, as more and more capillaries become occluded [5-7]. Appreciable increases in capillary nonperfusion/degeneration are strongly predictive of (and likely causally related to) progression to the advanced, neovascular stages of retinopathy in patients. Selective loss of pericytes is also common in DR, but perfusion can continue in capillaries missing only some pericytes.

Microaneurysms, or dot hemorrhages, are focal dilations of individual retinal capillaries, and are detectable ophthalmoscopically in diabetic patients. Increasing numbers of microaneurysms have a strong predictive value with respect to the progression of retinopathy [8,9]. Signs of advanced NPDR include venous beading and loops, intraretinal microvascular abnormalities, and large areas of capillary nonperfusion (as assessed using fluorescein angiography).

PDR is observed to develop in areas adjacent to extensive vascular nonperfusion, which are presumably ischemic [10,11]. This advanced stage of DR is characterized by the appearance of new, fragile, and fenestrated retinal blood vessels that penetrate the inner limiting membrane and enter the vitreous (i.e., preretinal neovascularization). These delicate vessels hemorrhage easily. The resulting pooling of blood in the vitreous will reduce in the amount of light reaching the retina and potentially decrease vision. Perhaps more importantly, fibrovascular scar tissue can form around the neovascularization, producing retinal tears and tractional detachment with subsequent blindness.

#### 1A2. Neuroglial pathology

Diabetes can also damage nonvascular cells of the retina. In autopsy samples, retinal ganglion cells are lost, at least in part via apoptosis [12-19]. In vivo scanning laser polarimetry or optical coherence tomography (OCT) studies have also measured a thinning of the nerve fiber layer in some diabetic patients [20-26]. It is not yet known if ganglion cell loss in diabetic patients is severe enough to contribute to impaired vision.

#### 1A3. Retinal thickness

OCT studies have also identified retinal thickening and serous retinal detachment in some diabetic patients [27-31]. However, some authors report that foveal thickness is similar among diabetics and nondiabetics when data are controlled for age, race, and sex [32].

#### 1A4. Permeability and edema

In early NPDR, apparent focal increases in vascular permeability (as assessed by focal accumulation of fluorescein near retinal vessels) are clearly associated with well defined microaneurysms [33-35]. Diffuse accumulation also occurs, but is not specifically associated with microaneurysms. These findings do not rule out an alternative possibility that a functional defect leading to the impaired removal of fluorescein contributes to the accumulation of the contrast agent as well as to the edema [36,37]. Baseline fluorescein accumulation in patients with DR was predictive of progression to photocoagulation for clinically significant macular edema [38].

#### 1A5. Glial activation

Induction of glial fibrillary acidic protein (GFAP) is a marker of glial activation, and upregulation of this protein occurs in Muller cells from the retinas of diabetic patients [39]. Activation of macro- and microglia occurs in the retinas of diabetic patients, but is of unknown significance [39-43]. Vascular endothelial growth factor, which is strongly implicated in retinal neovascularization and permeability in diabetes, is produced in retinal Muller cells [40].

### 1B. Characteristic functional defects

The above catalog of structural lesions, while critical in the clinical management of DR, provides little insight into the underlying mechanisms or pathophysiology of the disease, especially during its emerging, clinically silent phase. The pathophysiology of DR has also been extensively studied to better understand the effects of diabetes on the retina. These investigations offer a different perspective on retinal disease caused by diabetes.

#### 1B1. Perfusion and autoregulation

Much research has been directed at measuring diabetes-induced alterations in retinal perfusion, although there has been substantial disagreement in the literature regarding whether perfusion is increased, decreased, or unchanged [44-48]. The use of multiple different methods for monitoring plasma flow and erythrocyte velocity and transit time has likely contributed to the disparate findings. Nevertheless, steps toward understanding these differences come with evidence that perfusion changes are linked to levels of hyperglycemia and increasing duration of diabetes, along with the severity of retinopathy [47,49]. Retinal perfusion abnormalities are not detectable in patients with tight glycemic control [50].

**Figure 2:**
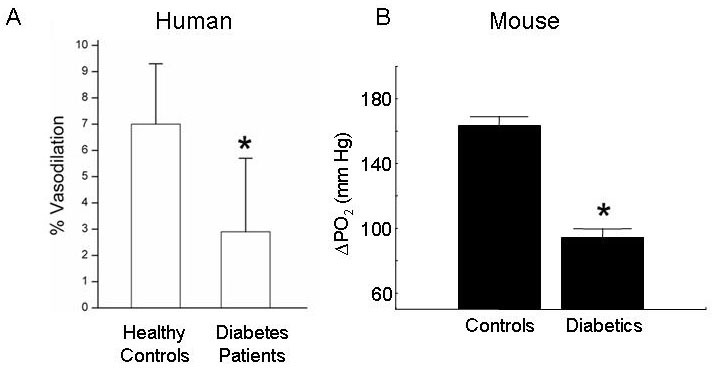
Diabetes induces defects in retinal vascular autoregulation in both humans and mice. **A**: The retinal arterial diameter response to stimulation with flickering light in age- and sex-matched controls and patients with type 1 diabetes mellitus. **B**: Plot of superior hemiretinal ΔPO_2_ during carbogen breathing indiabetic mice and sex-matched nondiabetic controls. * Significant difference (p<0.05). Figure A has been modified from Investigative Ophthalmology and Visual Science 50:4029–32, 2009, and Figure B from Diabetes (Copyright 2010 American Diabetes Association From Diabetes®, Vol. 53, 2004; 173–178. Modified with permission from The American Diabetes Association).

Measurements of retinal blood flow or perfusion have commonly been made in the unchallenged retina, but since the retinovascular system must constantly adapt to changes in blood pressure and metabolic demand, it is rarely found at steady-state. Dynamic studies of autoregulation, unlike perfusion measures [50], have robustly shown dysfunction of the vascular system in DR [51-54] (Figure 2). Importantly, retinal autoregulatory abnormalities can be detected in patients with tight glycemic control [50]. Whether or not the vascular autoregulation defects or retinal perfusion abnormalities in diabetes are of pathogenic importance is not yet known.

#### 1B2. Retinal function

Electrophysiological assessments of the retinal function in DR have been made using electroretinogram (ERG), multifocal ERG (mfERG), and visual evoked potential. Diabetes-induced dysfunction includes decreased b-wave amplitude, reduction of oscillatory potential amplitude, and delay in oscillatory potential latency [55-64]. Changes in ERG and mfERG have been used to predict progression of the retinopathy [55-58,65]. ERG and visual evoked potential integrate the response across the whole retina and do not allow the detection of localized abnormalities. Seemingly more useful is mfERG, which allows for functional assessment of multiple small areas of the central retina. Importantly, mfERG studies have been able to analytically predict the retinal locations of new nonproliferative DR development [65]. Another noninvasive method for analytically evaluating intraretinal function with good panretinal spatial resolution involves the measurement of retinal ionic regulation using manganese-enhanced magnetic resonance imaging (MEMRI) [66]. A Food Drug Administration (FDA)-approved manganese-based contrast agent (Teslascan) is beginning to be used to assess clinical applications of MEMRI in DR [67]

Diabetes also negatively impacts psychophysical processes related to vision, such as contrast sensitivity, color vision, and dark adaptation rates [68-71]. The reduction in contrast sensitivity in patients with diabetes, despite normal visual acuity, has been well documented [72,73], and significant losses of contrast sensitivity have been observed in patients with insulin-dependent diabetes mellitus with no evidence of retinopathy when compared with nondiabetic controls [74-81]. Spatial frequencies in the mid to high range are especially sensitive to diabetes [74,76,77,79,81]. These defects can occur before overt evidence of retinopathy is present [82]. Such deficits are not necessarily retina-specific, and could reflect changes anywhere in the visual system (from anterior chamber to visual cortex and higher central processing aspects in the brain). Diabetic patients take longer to dark adapt than age-matched controls [68]; this prolongation is likely caused by alterations in the photoreceptor/pigment epithelial complex.

## 2. Mice as models of diabetic retinopathy

A variety of different animal species (including primates, dogs, cats, pigs, rats, and mice) have been used to investigate the pathogenesis of retinopathy. The advantages and disadvantages of those species have been summarized elsewhere [2], and here we will focus only on mice, which offer considerable promise as models due largely to the availability of molecular tools to investigate the pathogenesis of the disease. To date, the effects of diabetes on the development of retinopathy in mice have been studied only in a small number of strains, so the following summary likely will need to be amended in the future.

### 2A. Characteristic structural changes

#### 2A1. Vascular pathology

Over a period of 6–9 months of diabetes, several strains of mice (including the C57Bl/6J, Ins2Akita, db/db, KK.Cg-Ay/J(KKAY), and Non-Obese Diabetic strains) develop similar microvascular changes of the retina that are consistent with the early clinical stages of NPDR (capillary degeneration, pericyte loss, thickening of the capillary basement membrane) [83-88]. C57Bl/6J mice made diabetic with streptozotocin begin to show capillary degeneration and pericyte loss histologically at about six months after the onset diabetes (Figure 1); these lesions become more numerous with increasing duration of the diabetes [88-90]. Males of the Ins2^Akita^ strain develop a spontaneous type 1 diabetes (females are less severely affected), with subsequent vascular histopathology [85] that is comparable in severity and progression rate to that in streptozotocin-treated C57Bl/6 mice. Likewise, genetically diabetic db/db mice (type 2 diabetes) develop strand-like and degenerate capillaries, as well as an increase in the ratio of endothelial cells to pericytes (interpreted as a loss of pericytes) relative to nondiabetic controls [87,91]. Crossing db/db mice with apolipoprotein E–deficient mice results in both hyperglycemia and hyperlipidemia [86], and these crossed mice exhibit accelerated degeneration of the retinal capillaries and pericytes compared with littermate controls [86]. The KK mouse strain exhibits pericyte ghosts, degenerate capillaries, and occasional microaneurysms between 20 and 64 weeks of age [92]. Thus, diabetic mice develop a similar vascular histopathology at prolonged progression rates to patients with early DR.

Genetically modified diabetic mice, however, can develop these structural lesions at an accelerated rate. For example, diabetic mice deficient in endothelial nitric oxide synthase are reported to have an earlier onset and increased number of acellular capillaries, sustained gliosis, and increased capillary basement membrane thickness than that reported for diabetic C57BL/6 mice [93]. In another example, KKAY mice have changes in their retinal capillaries after only three months of diabetes [84]. The changes reported were relatively nonspecific (endothelial hyperplasia, basement membrane thickening, and some edema and vacuolar degeneration of capillary cells), whereas none of the more characteristic lesions of DR (microaneurysms, capillary degeneration, pericyte loss, etc.) were discussed. Since the lesions detected were present after only short durations of diabetes, it is possible that they might be unrelated to diabetes.

Microaneurysms comparable to the saccular capillary lesions characteristic of diabetes in patients are not robustly reported in C57Bl/6 or Ins2^Akita^ mice [85,88,89,94]. Some KK mice have been reported to show occasional microaneurysm-like vascular abnormalities in old animals [92].

Thickening of the vascular basement membrane has been detected in retinal capillaries in mice with chemically induced [88,95] and spontaneous diabetes [96-98] after about six months diabetes. This change is not specific for the retina (as it is also found in kidney, brain, and other sites [99,100]), or even for diabetes [101].

A common criticism of the mouse model of DR is that it apparently does not develop preretinal neovascularization or other advanced lesions (retinal edema, hemorrhages, microaneurysms) of retinopathy [94,102]. The relatively short duration of diabetes (most studies are <1 year) and mouse lifespan, as well as the resulting modest extent of capillary degeneration that develops during this short interval, are probable reasons that neovascularization has not been observed in these models.

Nevertheless, some reports have claimed finding retinal neovascularization. Fifteen-month-old db/db mice showed increased density of retinal capillaries in the inner nuclear layer, which was interpreted as evidence of vessel proliferation [87]. However, these vessels did not extend into the vitreous body. J129sv/B16 mice diabetic for four months have been reported to develop neovascularization; the claimed new vessels were demonstrated by injecting a contrast medium (black ink) into the vascular lumen [103]. The “neovascularization” was poorly demonstrated, and could possibly be increased retinal vascular density within the retina instead.

#### 2A2. Neuroglial pathology

Neuronal cells of the retina also are affected by diabetes, resulting in dysfunction and even degeneration of some neuronal cells in humans and rats. Findings in mice, however, are more controversial, and have not necessarily been in agreement with findings in rats [88,104-107].

The spontaneous development of diabetes resulted in loss of cells in the ganglion cell layer in Ins2^Akita^ mice [85,108]. After 5–6 months of hyperglycemia, there was a significant reduction in the number of cell bodies in the retinal ganglion cell layer, which was accompanied by a significant reduction in the thickness of the inner plexiform layer in these animals. By crossing Ins2^Akita^ with mice that express fluorescent proteins under the regulation of the Thy1 promoter, Gastinger et al. [109] demonstrated that diabetes caused 16% depletion of ganglion cells from the peripheral retina, but not from the central region. Dopaminergic and cholinergic amacrine cells are also lost from the retina in diabetes [110].

The C57Bl/6J mouse strain has also been evaluated for diabetes-induced neurodegeneration in the retina. Some investigators reported a 20% to 25% reduction in cells of the ganglion cell layer of the retina at as early as 14 weeks of diabetes [104,107,111]. Others did not detect any ganglion cell loss at diabetes durations of up to one year [88,105,106,112]. This difference between findings remains unexplained. Fifteen-month-old db/db mice were reported to have increased apoptosis of retinal ganglion cells and other cells of the neural retina [87]. After one month of diabetes, the numbers of apoptotic cells in the retinal ganglion cells and inner nuclear layers were significantly greater in the diabetic KKAY mice than in the control group, and the rate of cell death increased with duration of diabetes [84].

#### 2A3. Retinal thickness

Studies of diabetic mice to date have shown thinning of the retina [85,90], in contrast to the retinal thickening seen in diabetic patients with retinal edema. The relative thickness of retinal layers has been found to be reduced in Ins2^Akita^ mice after 22 weeks of hyperglycemia [85], and in some (but not all) studies of C57Bl/6 mice made diabetic using streptozotocin [88,90,104,113]. As diabetic C57Bl/6 mice aged, MEMRI-derived measurements of retinal thickness in vivo decreased in a linear fashion [66].

#### 2A4. Permeability

A variety of techniques measuring accumulation of material from plasma in the neural retina of diabetic mice have been investigated to assess permeability [85,89,114-118]. Such accumulation seems diffuse in nature, and focal defects have not been reproducibly described in diabetic mice. Importantly, interpretations of techniques involving such tracer accumulation have not been validated in terms of the permeability surface area product (the gold standard metric for assessment of vascular leakage). Furthermore, whether or not the detection sensitivity and “lesion-type” (i.e., paracellular versus intracellular path defects) sensitivity of these assays are similar has not been established, making comparisons between laboratories/methods uncertain. Despite the indication of increased permeability in diabetes, edema has not been demonstrated in the retina of diabetic mice based on retinal thickness measurements (see section 2A3) [66,90]. Diabetic endothelial nitric oxide synthase^−/−^ mice exhibit accelerated and more severe retinal vascular permeability than age-matched diabetic control mice [93].

#### 2A5. Glial activation

Diabetes has not been found to result in upregulation of GFAP in Muller glial cells in retinas of C57Bl/6 mice [88,105] or Ins2^Akita^ diabetic mice [85]. In contrast, db/db diabetic mice were reported to show GFAP induction in diabetes, and this glial activation was inhibited in animals lacking aldose reductase [87]. Activation of microglia (based on shape change) also occurs in the retinas of diabetic mice [106], but is of unknown significance.

### 2B. Characteristic functional defects

#### 2B1. Perfusion and autoregulation

Only a few studies have been performed in diabetic mice [119-121], but there is controversy over changes in steady-state retinal perfusion. Nonetheless, diabetic mice demonstrate robust alterations in retinal autoregulatory ability in response to a provocation, similar to the defect reported in patients and other experimental models [119-123] (Figure 2). Correction of the retinal autoregulatory defect in diabetic mice is tightly linked with normalization of biochemical abnormalities, and is predictive of subsequent treatment efficacy [90,123,124].

#### 2B2. Retinal function

The primary means of assessing visual function in the mouse has been using electrophysiology methods such as ERG [86,90,125,126]. These ERG data provide evidence for dysfunction of both the outer retina and inner retina in DR. However, because the ERG measures a summed response from the entire retina, the relationship between such early dysfunction and later vascular histopathology remains unclear.

Assessment of intraretinal function in diabetes using MEMRI has also been reported, and genetic modifications that inhibited development of the vascular lesions of DR have been found to normalize the MEMRI response [66], suggesting a link between these events.

A method of assessing visual processes themselves (contrast sensitivity and visual acuity) in mice has recently become available [127-129] via measurement of the optokinetic response. Using this method, diabetes-induced reductions in contrast sensitivity and visual acuity have been demonstrated in mice ( [130], Kern et al. submitted, and Berkowitz et al. submitted).

## 3. Robust endpoints of retinopathy for use in diabetic mice

DR consists of a spectrum of retinal structural and functional lesions. Since DR is a composite of several different lesions, the presence of any one type of lesion is not sufficient to claim that it is present, nor is the correction of any one lesion sufficient to claim that the retinopathy has been inhibited. The fact that a particular species or strain does not develop the full spectrum of those lesions of retinopathy is obviously unfortunate, but likely is due, at least in part, to important differences between humans and rodents in the duration over which they are exposed to diabetes. The lesions which do develop in the animal models are characteristic of the early stages of retinopathy, and are worthy of continued study to identify promising new therapeutic targets at which to inhibit progression of the retinopathy to more advanced and clinically important stages (Table 1).

**Table 1:** Summary of structural and functional lesions in diabetic mice.

**Lesion**	**Reproducibly detected**	**C57Bl/6**	**Ins2Akita**	**db/db**	**Methods**	**When first detected (approx)**
Capillary degeneration	Yes	[88-90,113,131]	[85,134]	[86]	vessel digests	6 months
Defects in vascular autoregulation	Yes	[123,124]			MRI	4 months
Increased vascular permeability	Yes*	[85,89,115-117,132,133]	[85]		Evans blue, FITC	2 weeks –11 months
Retinal microaneurysms	Rare	[94]			vessel digests	
Capillary basement membrane thickening	Modest	[88]		[96]	ultrastructure	6–15 months
Retinal edema, retinal thickening	No	[66,90]			cross-sections, MRI	
Pre-retinal neovascularization	No	[94]			cross-sections, wholemounts	
Electrophysiological alterations	Yes	[90,106,126]		[86]	ERG, VEP	3 weeks
Intraretinal ion dysregulation in dark adaptation	Yes	[66]			MEMRI	3 weeks
Neuroglial apoptosis or remodeling	No	[88,104,105]	[109,110]	[87]	TUNEL, caspase-3	1–2 months
Loss of retinal ganglion cells	No	[88,104,105]	[85,108]	[87]	cross-sections	4–6 months

^*^Different methods do not all agree on the rate at which the defect develops.

Characteristics of DR in patients that have not been reproducibly replicated to date in diabetic mice are preretinal neovascularization, saccular microaneurysms, retinal hemorrhage, and retinal thickening due to edema.

Nevertheless, several abnormalities regarded as important in the development of DR (or at least characteristic of it) in humans have been robustly identified in diabetic mouse models. These are the early diabetes–induced degeneration of retinal capillaries (Figure 1), loss of capillary pericytes and neuroglia, impairment in vessel autoregulation (Figure 2), and deterioration of nonvascular retinal function. The capillary degeneration/nonperfusion seems to be especially important, because it is found in patients with NPDR and is clinically linked to the eventual development of PDR. A variety of validated techniques have been used to assess the capillary degeneration and the results of using different methods to isolate or view the retinal vessels (trypsin digest method, elastase method, whole-mount immunohistochemistry) have yielded similar conclusions. In contrast, a range of more or less validated (against standard physiologic methods) techniques have been use to, for example, evaluate retinal blood flow, but with less agreement between the methods. Thus, it is unclear if such disagreements are methodological or biologic in nature. Better attempts to validate and improve the physiologic accuracy of a chosen technique should help in addressing this issue.

In addition to the “standard” techniques that have been used to demonstrate changes in diabetic patients and rodents, new techniques (including adaptive optics, OCT, oxygenation mapping techniques, and MRI-based methods) are offering exciting new and potentially noninvasive ways to assess these (and other) DR lesions in the future.
